# The Neurochemical Orchestra of the Runner’s High: A Narrative Review of Neuromodulatory Mechanisms with a Focus on Endocannabinoids

**DOI:** 10.1177/10738584261440907

**Published:** 2026-05-07

**Authors:** Michael Siebers, Deborah Canales-Romero, Johannes Fuss

**Affiliations:** 1 Institute of Forensic Psychiatry and Sex Research, Center for Translational Neuro- and Behavioral Sciences, University of Duisburg-Essen, Essen, Germany; 2 Clinic of Forensic Psychiatry, Center for Translational Neuro- and Behavioral Sciences, University of Duisburg-Essen, Essen, Germany

**Keywords:** runner’s high, neuromodulation, endurance exercise, endocannabinoids, β-endorphins, dopamine, serotonin, brain-derived neurotrophic factor (BDNF), noradrenaline, adrenaline, leptin

## Abstract

Endurance exercise can induce a transient euphoric state known as the *runner’s high*, characterized by euphoria, anxiolysis, hypoalgesia, and a subjective sense of flow. Traditionally, this phenomenon has been interpreted within an “either–or” framework, attributing its emergence primarily to β-endorphins or, more recently, to the endocannabinoid (eCB) system. The aim of this narrative review is to move beyond this dichotomy and to present an integrative neuromodulatory perspective on the runner’s high, with a focus on the eCB system as a central coordinating mechanism. We integrate evidence from human and animal studies showing that circulating eCBs reliably increase during moderate-intensity endurance exercise and are consistently associated with core affective features of the runner’s high. At the same time, we consider the complementary roles of other neuromodulators: β-endorphins primarily regulate pain and stress responses; brain-derived neurotrophic factor supports neuroplasticity; serotonin and dopamine modulate mood and motivation; noradrenaline and adrenaline facilitate arousal; and leptin links the metabolic state to movement and motivation. Overall, the runner’s high appears to reflect an orchestrated neuromodulatory response in which the eCB system occupies a prominent integrative role.

## Introduction

Physical exercise modulates brain function and plays a significant role in promoting mental health (Singh et al 2023). Beyond general mood enhancement, endurance exercise can induce a transient affective state known as the *runner’s high*. This state is commonly described as involving euphoria, reduced anxiety, diminished pain perception, and a sense of sedation (Dietrich and McDaniel 2004). Some individuals also report altered time perception and a state of flow, marked by an effortless urge to keep exercising (Dietrich and McDaniel 2004). However, not all individuals report experiencing a runner’s high. In 2 studies involving nonprofessional athletes, approximately 70% stated that they had experienced a runner’s high at least once (Hinton and Taylor 1986; Siebers et al 2021). The highly subjective nature of the runner’s high makes it difficult to establish a standardized definition, as its intensity and manifestation vary considerably across individuals and are often subtler than altered states induced by psychoactive substances. Moreover, retrospective self-reports are prone to bias and variability in interpretation, complicating its classification within conventional scientific constructs.

Our previous work primarily addressed whether the runner’s high is mediated by endocannabinoids (eCBs) or endogenous opioids. In a mouse model, we found that 2 hallmark features of the runner’s high—anxiolysis and exercise-induced hypoalgesia—depend on intact eCB signaling rather than opioid signaling, as shown by the systemic blockade of both pathways (Fuss et al 2015). We subsequently translated these findings to humans in a randomized, double-blind, placebo-controlled treadmill study involving 63 participants (Siebers et al 2021). Despite pharmacologic opioid blockade with naltrexone, participants reported comparable euphoria and anxiolytic effects, as well as comparable ratings of runner’s highs. Furthermore, participants exhibited significant postexercise increases in circulating eCBs following running. These observations were independent of opioid signaling. A subsequent systematic review confirmed that exercise-induced eCB release represents a robust and reproducible finding across endurance modalities, while long-term training appears to reduce baseline eCB levels, suggesting adaptive regulation (Siebers et al 2023).

In the present narrative review, we therefore broaden our perspective from an exclusive “eCBs vs opioids” framework toward a more integrative “eCBs and other neuromodulatory systems” model of the runner’s high. We specifically examine how additional neuromodulators may contribute to the affective and perceptual features of endurance exercise and how these systems interact with eCB signaling. While the focus is placed on human studies, the limited accessibility of central neuromodulatory processes necessitates cautious reference to animal models, particularly when addressing mechanistic interactions among multiple neuromodulatory pathways. In this narrative review, we focus on the neuromodulators most often cited in the literature as mediators of the runner’s high.

## Endocannabinoids

**Figure 1.**
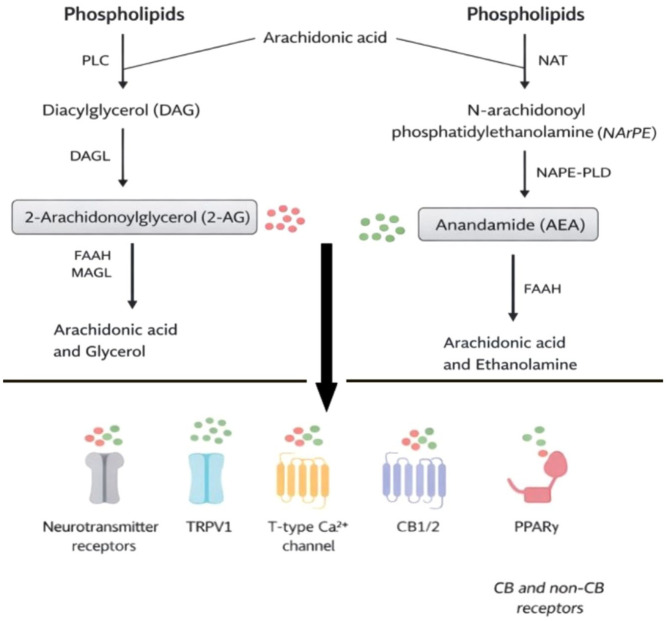
Schematic illustration of major cannabinoid and noncannabinoid receptor targets of the endocannabinoids anandamide (*N*-arachidonoylethanolamine; AEA) and 2-arachidonoylglycerol (2-AG). The endocannabinoid system is mainly composed of AEA and 2-AG. AEA is synthesized on demand from *N*-arachidonoyl-phosphatidylethanolamine by *N*-acyl-phosphatidylethanolamine-specific phospholipase D (NAPE-PLD) and primarily degraded by fatty acid amide hydrolase (FAAH). 2-AG is formed from diacylglycerol by diacylglycerol lipase (DAGL) and hydrolyzed by monoacylglycerol lipase (MAGL) as well as α/β-hydrolase domain-containing proteins 6 and 12 (ABHD6, ABHD12), yielding arachidonic acid and glycerol. Both endocannabinoids are synthesized from membrane-derived arachidonic acid. AEA and 2-AG act at cannabinoid receptors 1 and 2 (CB1, CB2) and at noncannabinoid targets, including transient receptor potential vanilloid 1 (TRPV1), T-type Ca²⁺ channels, peroxisome proliferator-activated receptor γ (PPARγ), and other neurotransmitter-related receptors. Colored dots indicate ligand–receptor interactions without implying binding affinity or signaling strength. NAT, N‑acyltransferase ; PLC, phospholipase C.

The eCB system consists primarily of anandamide (*N*-arachidonoylethanolamine; AEA) and 2-arachidonoylglycerol (2-AG; Hillard 2018). AEA is synthesized on demand from *N*-arachidonoyl phosphatidylethanolamine, a member of the *N*-acyl-phosphatidylethanolamine family, predominantly by *N*-acyl-phosphatidylethanolamine-specific phospholipase D (Ueda et al 2013). It is primarily degraded by fatty acid amide hydrolase (FAAH; Reggio 2010). In contrast, 2-AG is generated from diacylglycerol by diacylglycerol lipase and hydrolyzed by monoacylglycerol lipase as well as α/β-hydrolase domain-containing proteins 6 and 12 (ABHD6 and ABHD12). Beyond cannabinoid receptors 1 and 2 (CB1 and CB2), AEA engages noncannabinoid targets such as transient receptor potential vanilloid 1 (TRPV1) channels, while AEA and 2-AG can activate peroxisome proliferator-activated receptors (PPARs), particularly PPAR-α and PPAR-γ (Tantimonaco et al 2014; O’Sullivan 2016; Matei et al 2023; Figure 1). AEA shares biosynthetic and degradative pathways with palmitoylethanolamide, oleoylethanolamide, and stearoylethanolamide, members of the *N*-acyl ethanolamine family, which are lipid mediators derived from *N*-acyl-phosphatidylethanolamines and co-regulated by FAAH (Tantimonaco et al 2014). eCBs are synthesized on demand from membrane phospholipids containing arachidonic acid, which serve as a readily available precursor pool within the lipid membrane (Tallima and El Ridi 2018). Due to their lipophilic nature, they easily cross the blood-brain barrier (BBB; Dietrich and McDaniel 2004). The eCB system plays crucial roles in immune modulation, mood regulation, memory processing, neurodevelopment, and appetite control (Hillard 2018; Watkins 2019).

Systematic reviews consistently report increases in AEA during endurance exercise, whereas 2-AG is often not significantly elevated, suggesting distinct physiologic roles (Desai et al 2021; Siebers et al 2023). A functional difference between AEA and 2-AG may lie in their interaction with TRPV1 receptors and nociceptive pathways. AEA activates TRPV1 receptors on nociceptive C- and Aδ-neurons, which may modulate 2-AG signaling and downstream nociceptive pathways (Matei et al 2023). TRPV1 receptors and their downstream signaling cascades play a central role in hyperalgesia, persistent pain states, inflammatory processes, neurogenesis, and anxiety, linking AEA-mediated TRPV1 activation to the affective and nociceptive facets of prolonged endurance exercise (Matei et al 2023). In contrast, 2-AG is involved in postexercise inflammatory resolution and tissue repair, acting via monoacylglycerol lipase–dependent conversion to arachidonic acid and subsequent prostaglandin synthesis, particularly in peripheral tissues (Cao et al 2013). Its elevation after exercise may therefore indicate the initiation of regenerative processes, including membrane repair and immune cell recruitment (Cao et al 2013; Jiang et al 2023). With increasing exercise duration and intensity, physiologic regulation may progressively shift toward these reparative, inflammation-linked pathways. Taken together, this supports the view that AEA primarily mirrors the subjective and motivational components of endurance performance, whereas 2-AG reflects more fundamental homeostatic responses associated with cellular stress, inflammation, and recovery (Matei et al 2023).

**Figure 2.**
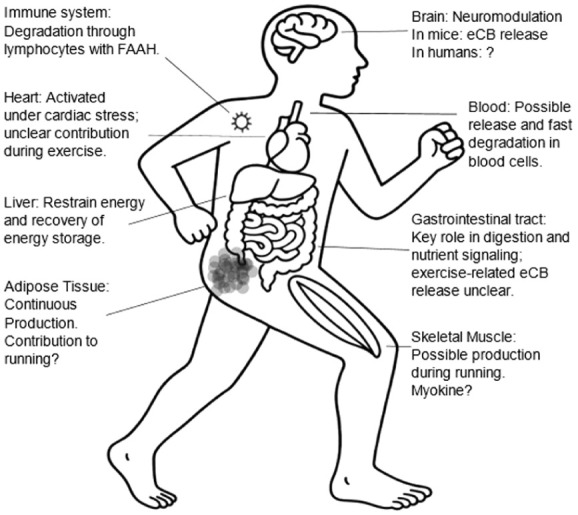
Potential sources and pathways of eCB release during endurance exercise. Schematic overview of organs potentially involved in the production, release, and degradation of eCBs during endurance running. Skeletal muscle is considered a key peripheral contributor to circulating eCBs, particularly anandamide, while immune cells and blood cells may regulate degradation. The gastrointestinal tract, despite a functional eCB system, likely plays a minor role during exercise due to reduced blood flow. Central effects in the brain are hypothesized to arise from local synthesis and peripheral transport. The precise origin and tissue-specific contribution of eCBs during exercise remain to be clarified. eCB, endocannabinoid; FAAH, fatty acid amide hydrolase.

Although several studies found that eCB levels increase during endurance exercise, the precise tissue origins remain unclear (Fuss et al 2015; Desai et al 2021; Siebers et al 2023; Matei et al 2023). Skeletal muscle is often discussed as a primary source due to its mass and metabolic activity during running (Figure 2), while degradation of eCBs may occur in the vasculature (Matei et al 2023; Di et al 2024). Immune cells such as lymphocytes appear to regulate eCB levels via FAAH expression, potentially under the influence of interleukin 6 (Gasperi et al 2014). Interleukin 6 is a myokine known for its shift from proinflammatory to anti-inflammatory effects during sports (Docherty et al 2022). Whether eCBs can be regarded as a myokine and how they interact with interleukins, needs further research. Adipose tissue constitutively produces eCBs, and its role in acute release during exercise is debated, especially since baseline levels of AEA and 2-AG tend to decrease after endurance training over several weeks (Engeli et al 2005; Siebers et al 2023; Di et al 2024). The heart can generate eCBs in response to pathologic stress, but its contribution during endurance exercise is not well established (Hiley 2009). The liver may participate in regulating eCB availability through lipid metabolism, and the gastrointestinal tract possesses all necessary components for eCB synthesis, although its role during exercise may be limited due to reduced blood flow (Qamar and Read 1987).

To date, no study has identified exercise-induced eCB activity using radiotracer imaging techniques such as positron emission tomography (PET). Some PET tracers targeting components of the eCB system have been developed and applied in humans (Burns et al 2007; Terry et al 2010; Normandin et al 2015). However, several studies in mice showed an impact of exercise on eCB brain activity, especially in the striatum (De Chiara et al 2010), hippocampus (Hill et al 2010; Ferreira-Vieira et al 2014), periaqueductal gray matter (Galdino et al 2014), and forebrain (Fuss et al 2015). These regions are implicated in reward processing, memory, anxiety regulation, and pain modulation—functions that are relevant to endurance exercise and runner’s high–related outcomes. A rodent study reported significantly elevated levels of AEA and a trend toward increased 2-AG in hippocampal tissue after 8 d of voluntary wheel running (Hill et al 2010). These changes were not observed in the prefrontal cortex. CB1 knockout mice (CB1⁻/⁻) exhibit approximately 30%–40% less voluntary wheel-running activity than wild type controls, spending less time running and reaching lower running speeds (Dubreucq et al 2010). A recent study showed that chronic stress increases the permeability of the BBB to inflammatory molecules, a process mediated by CB1 receptors on astrocytes (Dudek et al 2025). Mice with higher CB1 receptor expression exhibited reduced anxiety and fewer depressive-like symptoms. Overall, the relative contribution of each tissue to the circulating eCB pool during endurance activity remains unresolved, highlighting the need for further targeted research, particularly in human models, to clarify the dynamics of production, release, and degradation across organ systems.

The first eCBs were identified in the 1990s under the leadership of Raphael Mechoulam, following the isolation of THC (tetrahydrocannabinol) from *Cannabis sativa* in the 1960s (Gaoni and Mechoulam 1964; Mechoulam et al 2014). Thus, the eCB system was not yet discovered when the runner’s high was first described. After the discovery of the eCB system, researchers investigated its role in the runner’s high. Sparling et al (2003) first observed eCB increases after running and a less pronounced effect after cycling. Dietrich and McDaniel (2004) categorized the runner’s high characteristics and named eCBs as key mediators. In a pivotal study, Raichlen et al (2012) found that only endurance-adapted species, such as humans and dogs, showed significant eCB increases during running, whereas animals with low endurance capacity (eg, ferrets) did not. The authors interpreted these findings as suggesting that eCB signaling may have evolved to facilitate endurance running. Our own research confirmed eCB elevation after 45 min of treadmill running and walking (Siebers et al 2021). However, euphoria and anxiolysis—key runner’s high features—were observed only after running. Three systematic reviews on exercise-induced eCB release found consistent eCB increases during endurance exercise, correlating with anxiolysis and euphoria (Desai et al 2021; Siebers et al 2023; Schaumberg et al 2025). A study recently found significantly increased AEA but not 2-AG levels after an ultramarathon of up to 100 km (Dalle et al 2024).

Several factors have been identified as optimal for maximizing eCB production under laboratory conditions. The highest eCB levels are typically produced below the anaerobic threshold (Raichlen et al 2013). Running elicits the strongest eCB response, followed by cycling (Sparling et al 2003; Siebers et al 2023). Training duration should be at least 20 min, with peak mood benefits occurring after 30 to 35 min (Berger and Motl 2000; Ekkekakis et al 2011). Prior experience with the exercise may be essential for developing a runner’s high (Weiermair et al 2024). Furthermore, training in nature seems to be beneficial for some individuals (Feuerecker et al 2012; Weiermair et al 2024), with eCB levels peaking immediately postexercise (Siebers et al 2023).

Taken together, eCBs appear to be crucial for endurance performance and the emergence of the runner’s high. Experimental studies have shown their influence particularly on key features such as euphoria and anxiolysis. Current evidence suggests that eCBs may act as central mediators of the runner’s high, interacting with other neuromodulatory systems.

## β-Endorphin

The opioid system includes peptides (endorphins, enkephalins, dynorphins) and their receptors (μ, δ, κ) that are widely distributed in the central and peripheral nervous systems (Stein 2016). The μ-opioid receptor is the primary receptor responsible for reward and pain relief, while κ-opioid receptors are linked to dysphoria and stress adaptation (Lutz and Kieffer 2013). Furthermore, there is evidence that β-endorphin is an anti-inflammatory substance and a regulator of stress relief (Pilozzi et al 2020). Proopiomelanocortin is cleaved into β-endorphins and adrenocorticotropic hormone (ACTH) by the prohormone convertases PC1 and PC2. ACTH, in turn, is processed into cortisol, which is well known as a stress hormone. ACTH and β-endorphins are stored and released together in secretory vesicles (Gianoulakis 1998).

The first descriptions of a runner’s high in scientific studies appeared in the late 1970s (Black et al 1979; Sachs and Pargman 1979; Sachs and Buffone 1984). Just a few years earlier, John Hughes and Hans Kosterlitz discovered the first enkephalins, which bind to opioid receptors, and 1 year later, β-endorphins were identified (Hughes et al 1975; Li and Chung 1976). Subsequently, endorphins were widely associated with feelings of happiness, both in society and within scientific discourse. Therefore, numerous studies attempted to clarify the role of endorphins in exercise-induced euphoria, yielding contradictory results. While some studies reported an increase in peripheral β-endorphins during endurance exercise, the findings regarding mood modulation remained inconsistent (Markoff et al 1982; Farrell et al 1986). Several studies found that blocking the opioid system did not alter the subjective experience of endurance exercise (Markoff et al 1982; Farrell et al 1986; Siebers et al 2021). However, some studies found analgesic effects and euphoria during endurance exercise that were associated with endorphin activity (Harber and Sutton 1984; Boecker et al 2008; Goldfarb 2013). Importantly, peripheral β-endorphins are hydrophilic peptides and do not readily cross the BBB, which raises doubts about their direct role in central nervous system regulation during exercise-induced euphoria (Dietrich and McDaniel 2004; Dishman and O’Connor 2009).

A review linked β-endorphin release to exercise intensity and duration, indicating that higher intensity and longer exercise durations result in greater β-endorphin production (Goldfarb 2013). An intensity >60% VO₂max has been suggested to trigger β-endorphin release (Goldfarb and Jamurtas 1997), although this may vary by individual factors and training levels. Furthermore, graded and short-term anaerobic exercise elevates β-endorphin levels, which are linked to rising lactate concentrations once the anaerobic threshold is surpassed (Goldfarb 2013). During steady-state endurance exercise, β-endorphin rises only after ~1 h, then it increases exponentially (Schwarz and Kindermann 1992). β-endorphin and ACTH are released simultaneously, followed by a delayed cortisol response (Schwarz and Kindermann 1992). For longer durations, β-endorphins may not coincide with lactate accumulation (Goldfarb 2013). Interestingly, β-endorphins are also increased in resistance exercise with a greater increase in studies with higher intensity and workload (Goldfarb 2013).

As opioids cannot cross the BBB (Boehler et al 2017), neuroimaging studies provided further insights into the central opioid response to endurance exercise. Boecker et al (2008) showed that 2 h of moderate-intensity endurance running (average heart rate, 144 ± 7 beats · min^-1^) resulted in significant opioid release in the prefrontal cortex and limbic system. In contrast, another study showed that 1 h of low-intensity cycling below the anaerobic threshold elicited opioid activation only in some individuals, with no statistically significant group-level effect (Saanijoki et al 2018a). High-intensity interval training, however, was found to trigger robust opioid release in frontolimbic regions involved in pain processing, reward, and emotional regulation, whereas moderate-intensity exercise did not produce significant changes (Saanijoki et al 2018b). Furthermore, more highly trained individuals exhibited greater acute opioid release following a maximal incremental cycling test, particularly in brain regions associated with reward and cognitive processing (Saanijoki et al 2022).

### eCBs and β-Endorphins in Human and Animal Studies

To date, studies investigating the synergistic effects between eCBs and opioids are scarce. By blocking the opioid system, our mice and human study indicated that the characteristic features of the runner’s high are primarily mediated by eCB signaling (Fuss et al 2015; Siebers et al 2021). However, several studies have found a synergistic interplay between the opioid and eCB systems in the regulation of reward, anxiety, and pain, particularly in the context of medical treatment and addiction (Zarrindast et al 2008; Befort 2015; Martínez-Rivera et al 2024). Martínez-Rivera et al (2024) indicated that increasing levels of the eCB 2-AG (via monoacylglycerol lipase inhibition) in mice blocked morphine-induced addictive behavior without impairing its analgesic effects. This effect was CB1 receptor–dependent and involved a reduction in dopamine release within the ventral tegmental area to the nucleus accumbens. These findings suggest that enhancing 2-AG signaling could help prevent opioid addiction while preserving effective pain relief. As hypoalgesia is a key component of the runner’s high, this interplay suggests a cooperative interaction between the eCB and opioid systems in mediating its analgesic effects.

Previous studies have consistently shown that CB1 receptor blockade can partly reverse morphine-induced analgesia, while the opioid antagonist naltrexone can partly inhibit THC-induced analgesia, supporting bidirectional interactions (Befort 2015). In a systematic review and meta-analysis, Nielsen et al (2017) reported synergistic effects in 17 of 19 preclinical studies, with the median effective dose of morphine being 3.6 times lower when co-administered with Δ⁹-THC (delta-9-tetrahydrocannabinol) as compared with morphine alone in rodents. Nevertheless, knockout studies indicate that the opioid and eCB systems retain independent analgesic pathways, as cannabinoid and opioid analgesia remains functional even in the absence of the other system (Befort 2015). At present, there is limited evidence for an interplay between eCBs and opioids during endurance sports, indicating a need for further investigation.

Taken together, while β-endorphins are predominantly released during high-intensity or long-duration exercise, they contribute to pain modulation and may influence mood, particularly under conditions of metabolic strain, elevated lactate, or prolonged physical stress. However, current evidence suggests that they are not the primary mediators of the runner’s high but rather part of a complex, interacting neuromodulatory network in which the eCB system plays a central role.

## Leptin

Leptin, discovered in the mid-1990s, is a hormone primarily synthesized by adipocytes (Watts et al 2022). Its main function is to regulate energy balance by signaling peripheral fat stores to the brain, promoting satiety and regulating body weight (Mendoza-Herrera et al 2021). While its primary source is adipose tissue, leptin is also produced in smaller amounts by the gastric wall, vascular cells, placenta, ovaries, liver, and skeletal muscle (Huo et al 2007; Bouassida et al 2010).

Several studies have investigated the conditions under which leptin levels decline in response to acute and chronic exercise, with partially divergent findings: acute reductions in circulating leptin appear to require prolonged or energetically demanding exercise, typically exceeding 60 min or ~800 kcal, and have been observed across different exercise modalities (Bouassida et al 2010). In contrast, other reviews suggest that during running, measurable acute leptin decreases occur only after extremely long distances, such as marathon-equivalent efforts (Alves et al 2022). Regarding chronic adaptations, regular exercise is associated with a modest but consistent reduction in baseline leptin levels, largely driven by decreases in body fat and potentially improved leptin sensitivity (Fedewa et al 2018). More recent data indicate that acute and chronic effects are modulated by nutritional status, with fasting combined with sufficient weekly exercise volume showing the strongest leptin-lowering effects (Fontana et al 2023).

Leptin has emerged as a potential biomarker related to exercise adaptation. Bobbert et al (2012) reported that lower leptin concentrations were associated with faster marathon times, suggesting a possible link between leptin levels and endurance performance. Conversely, markedly reduced leptin levels have been linked to overtraining syndrome (Joro et al 2017). More recently, Fontana et al (2023) proposed a critical threshold of 2 ng/mL, below which the risk of overtraining may increase. However, while low leptin may serve as a warning signal, it is not sufficient on its own to indicate overtraining (Armstrong et al 2022). This notion aligns with findings from exercise addiction research, where similarly diminished leptin levels have been observed, pointing to its relevance in compulsive exercise behavior as well (Lichtenstein et al 2015).

The extent to which leptin affects reward-driven movement remains a topic of debate. Fernandes et al (2015) found that leptin receptor signaling in dopamine neurons regulates voluntary wheel running in male mice. Inactivating STAT3 in the ventral tegmental area, a key reward-processing center, led to increased running, suggesting that leptin normally suppresses endurance exercise. Restoring STAT3 reversed this effect, while leptin injections into the ventral tegmental area reduced motivation for activity.

In contrast, a later study by the same group found no such effects in female mice (Fernandes et al 2021). STAT3 deletion did not affect feeding, locomotion, wheel running, or food preference, although the affected females showed heightened anxiety-like behavior and elevated corticosterone in response to stress. The authors hypothesized that sex differences in reward and anxiety regulation influence leptin’s role in the reward system.

**Figure 3.**
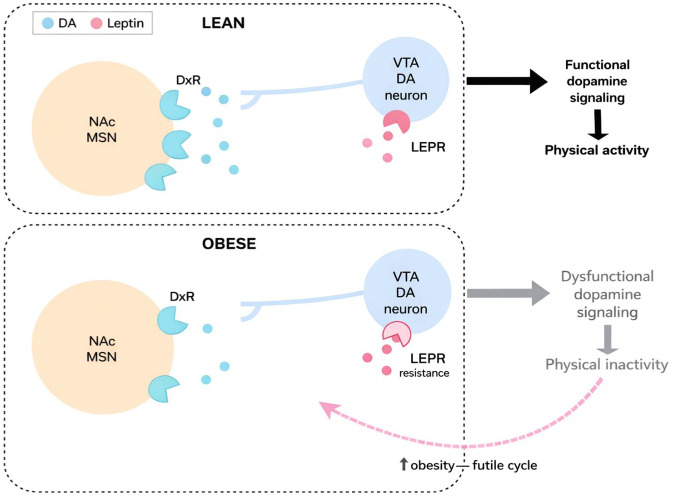
Leptin–dopamine (DA) interactions within the ventral tegmental area–nucleus accumbens (VTA–NAc) pathway and their role in physical activity regulation in lean vs obese states. Illustration adapted from Ruegsegger et al (2017), depicting the dopaminergic VTA → NAc pathway and the impact of leptin resistance on physical inactivity in obesity. Licensed under CC BY 4.0. (Top panel LEAN) In lean individuals, medium spiny neurons (MSNs) of the NAc express DA receptors, especially D2, at normal levels, allowing effective dopaminergic signaling. DA is released from neurons in the VTA, where functional leptin receptors (LEPRs) respond to circulating leptin. This supports motivation and regular physical activity. (Bottom panel OBESE) In obesity, medium spiny neurons in the NAc show reduced expression of D2 receptors, weakening the motivational response to DA. At the same time, high leptin levels lead to central leptin resistance, impairing LEPR signaling in VTA DA neurons and reducing DA release. This blunted reward signaling contributes to decreased movement and reinforces a cycle of physical inactivity and further obesity. DxR, dopamine receptor(s).

Leptin likely played a key role in evolutionary energy regulation (Denver et al 2011). Historically, energy storage was crucial for survival, driving individuals to seek food when levels were low while promoting rest when energy reserves were sufficient (Lieberman 2015). Humans evolved to conserve energy rather than actively resist inactivity, making leptin a potential driver of movement and rest. In modern times, obesity has become a growing health concern, with leptin resistance implicated as a contributing factor (Ruegsegger and Booth 2017). These authors described a vicious cycle, as demonstrated in Figure 3.

Likewise, and on the opposite side of the spectrum, anorexia-induced hyperactivity has been linked to the leptin system (Hebebrand et al 2019). Interestingly, a case report documented that hyperactivity ceased following metreleptin therapy in a female patient with anorexia nervosa (Gradl-Dietsch et al 2023).

### Leptin and eCB Interaction in Mice

As shown by Balsevich et al (2018), leptin suppresses food intake via an eCB-dependent mechanism involving increased activity of FAAH, a key enzyme in eCB degradation, and reduced hypothalamic AEA signaling in mice. The hypophagic effect is impaired in diet-induced obesity and further modulated by genetic variation, with carriers of the FAAH 385A allele exhibiting reduced leptin sensitivity and a heightened risk for obesity and related metabolic disorders. This draws a direct connection between the eCB system and leptin, which may also be relevant to neurometabolic adaptations during endurance exercise and warrants further investigation in the context of physical activity.

Although leptin’s involvement in the runner’s high is not yet fully understood, it is increasingly recognized as a modulator of movement motivation, with low levels potentially enhancing activity and high levels favoring rest.

## Brain-Derived Neurotrophic Factor

Brain-derived neurotrophic factor (BDNF) is a critical neurotrophin produced in the brain and peripheral tissues (Walsh et al 2015). Mature BDNF binds to the TrkB receptor and activates intracellular signaling pathways involved in neuroplasticity, neuronal survival, and learning; accordingly, BDNF is thought to contribute to exercise-related improvements in mood and cognition (Szuhany et al 2015). Its expression is linked to neuronal activity, particularly in brain regions such as the hippocampus, cerebral cortex, hypothalamus, and cerebellum (Murer et al 2001).

BDNF expression is upregulated during aerobic exercise via multiple molecular pathways (Dinoff et al 2016; Walsh and Tschakovsky 2018). While centrally produced BDNF is capable of crossing the BBB, peripheral tissues also contribute to circulating BDNF. This dual origin complicates the interpretation of changes in serum BDNF levels in humans (Murer et al 2001; Erickson et al 2012). Current evidence suggests that the brain is the primary source of circulating BDNF at rest and during exercise (Walsh and Tschakovsky 2018). Although BDNF may cross the BBB via a limited, saturable transport mechanism, this process remains controversial (Walsh and Tschakovsky 2018). Consequently, exercise-induced increases in circulating BDNF are generally interpreted as reflecting central release rather than peripheral BDNF entering the brain. However, there is evidence that peripheral and central BDNF levels seem to correlate (Szuhany et al 2015).

Even a brief session of 15 min of moderate-intensity aerobic exercise can elevate peripheral BDNF levels (Tang et al 2008). Higher intensity and longer durations appear to further enhance BDNF production (Schmolesky et al 2013). A significant increase is typically observed up to 10 to 15 min after the end of exercise (Tang et al 2008; Rojas Vega et al 2011). One study also found a positive correlation between lactate concentration and BDNF levels (Ferris et al 2007). In a review, Huang et al (2014) concluded that a single session of aerobic exercise, such as running or cycling for 20 to 90 min at 40% to 60% of VO₂max, increases BDNF levels. Meta-analyses confirm a significant increase in BDNF following acute exercise, with more pronounced effects after chronic training (Szuhany et al 2015; Dinoff et al 2016; Wang et al 2022; Schaumberg et al 2025). Interestingly, studies involving long-term physical exercise with larger sample sizes, female participants, individuals >60 y old, and aerobic exercise showed a more significant increase in BDNF levels (Wang et al 2022). This suggests that women and older individuals may particularly benefit from increased BDNF levels, potentially leading to improvements in cognition and mood.

Reduced BDNF levels have been associated with depression, anxiety, and acute and chronic stress (Murakami et al 2005; Porter and O’Connor 2022). The antidepressant effect of physical activity has been confirmed in meta-analyses, which found it to be comparable to, or even slightly more effective than, psychotherapy and pharmacotherapy (Singh et al 2023). In animal models, central blockade of BDNF prevented exercise-induced cognitive improvements (Vaynman et al 2004). It has been hypothesized that BDNF places the brain in a state of readiness for plasticity (Cotman et al 2007). Notably, moderate- to high-intensity exercise was associated with greater reductions in depressive symptoms than low-intensity exercise—an effect that may be partially mediated by BDNF (Singh et al 2023).

### BDNF and eCBs in Humans and Animals

There is growing evidence for an interaction between eCBs and BDNF. For example, Heyman et al (2012) found a significant correlation between eCB levels and BDNF during moderate to vigorous exercise in humans. The authors hypothesized that an increase in AEA might trigger the exercise-induced elevation of BDNF, thereby promoting neuroplasticity and antidepressant effects. Ferreira-Vieira et al (2014) showed that blocking CB1 receptors in rats abolished the exercise-induced increase in BDNF. Further evidence comes from Marin Bosch et al (2020), who confirmed that improvements in cognitive performance were observed only after moderate-intensity endurance exercise but not after high-intensity exertion in humans. Increases in AEA and BDNF were positively associated with memory enhancement, and functional magnetic resonance imaging revealed that hippocampal memory representations were modulated by these factors exclusively following moderate exercise (Marin Bosch et al 2020). Beyond cognition, AEA and BDNF have been implicated in anxiety and fear regulation. Crombie et al (2021) found that moderate-intensity endurance running promoted fear extinction in women diagnosed with posttraumatic stress disorder, an effect partially mediated by increases in AEA and BDNF.

In summary, BDNF is upregulated by endurance exercise given sufficient intensity and duration, with even stronger effects observed following regular training. Although higher exercise intensities may induce greater increases in BDNF, cognitive and affective benefits appear to follow an inverted U-shaped relationship, with moderate intensities producing the most consistent improvements (Ferris et al 2007; Marin Bosch et al 2020). It likely contributes to improved cognitive function, mood regulation, fear extinction, and stress resilience, possibly playing a central role in the neurobiological mechanisms underlying the psychological benefits of physical activity in conjunction with AEA levels.

## Serotonin

Serotonin (5-HT) is a monoamine synthesized primarily from the essential amino acid tryptophan via the enzymes tryptophan hydroxylase and aromatic amino acid decarboxylase. While it is widely recognized as a central neurotransmitter in the brain, about 90% to 95% of the body’s 5-HT is produced peripherally by enterochromaffin cells in the gastrointestinal tract, where it functions as a hormone regulating intestinal motility, vascular tone, and platelet aggregation (Berger et al 2009; Wei et al 2022). 5-HT plays a central role in regulating mood, motivation, and neurogenesis and modulating central fatigue, particularly during prolonged endurance exercise (Zimmer et al 2016; Basso and Suzuki 2017). Although 5-HT does not easily cross the BBB, 2 studies suggest the presence of a 5-HT transporter at the BBB, enabling selective transfer under specific conditions (Nakatani et al 2008; Young et al 2015). 5-HT metabolism in the brain is tightly regulated across spatial and temporal dimensions (Heijnen et al 2015). Supporting its role in exercise-induced antidepressant effects, animal studies have shown increased 5-HT concentrations in the cerebrospinal fluid and brain ventricles, along with enhanced firing rates of serotonergic neurons following physical activity (Chaouloff et al 1986; Ge and Dai 2020). Importantly, most peripheral 5-HT is stored in platelets, which cannot cross the BBB.

Exercise intensity appears to modulate 5-HT levels significantly: the most substantial increase was observed at 85% to 90% of maximal heart rate (anaerobic threshold) during 35 min of cycling (Zimmer et al 2016). These results are consistent with elevated 5-HT levels reported after ultramarathon events and under heat stress (Zhao et al 2015; Agrawal et al 2018). Notably, higher postexercise 5-HT levels have been associated with enhanced cognitive performance (Zimmer et al 2016).

5-HT is synthesized from tryptophan, which competes with branched-chain amino acids (BCAAs) for BBB transport (Heijnen et al 2015). During prolonged exercise, muscle uptake lowers BCAA levels, facilitating greater tryptophan entry and 5-HT synthesis. Thus, the tryptophan-to-BCAA ratio is proposed as a peripheral biomarker of central fatigue (Davis et al 1992; Melancon et al 2012). According to the central fatigue hypothesis, elevated brain 5-HT, alongside reduced dopamine, leads to fatigue and motivational decline (Davis et al 1992).

### 5-HT and eCBs in Human and Animal Studies

Notable similarities between the behavioral effects of 5-HT and eCB activity have been frequently reported, particularly in the regulation of emotional states, stress balance, cognitive functions, appetite, and sleep (Colangeli et al 2021). Serotonergic neurons have CB1 receptors that mediate retrograde synaptic modulation, particularly in the dorsal raphe nucleus (Haj-Dahmane and Shen 2011). Mice lacking CB1 receptors on serotonergic neurons exhibit increased anxiety-like behavior and reduced sociability (Häring et al 2015). However, studies exploring serotonergic–eCB system interactions in the context of human endurance exercise remain limited. Future research should aim to clarify the functional relationship between these systems in the context of physical activity.

In summary, 5-HT appears to contribute to the mood-enhancing and neuroplastic effects of endurance exercise, while excessive serotonergic activity may promote central fatigue under prolonged strain. The observed convergence between the eCB and serotonergic systems suggests a coordinated neuromodulatory network that may underlie the affective and motivational benefits of sustained aerobic activity.

## Noradrenaline and Adrenaline

Noradrenaline (norepinephrine) and adrenaline (epinephrine) are catecholamines, alongside dopamine, and form a key part of the sympathetic nervous system (Goldstein 2010). They mediate the body’s acute stress response by increasing heart rate, elevating blood pressure, and mobilizing energy substrates and priming the organism for the classic fight-or-flight response while enhancing alertness. Adrenaline acts primarily as a hormone secreted by the adrenal medulla, whereas noradrenaline serves as a neurotransmitter and a hormone, released by sympathetic nerve endings to regulate vascular tone and blood pressure (Goldstein 2010).

During physical exercise, catecholamines play a central role in energy metabolism, particularly by promoting glycogenolysis and facilitating the delivery of glucose to working muscles (Zouhal et al 2008). Zouhal et al (2008) reported that noradrenaline and adrenaline levels can rise 1.5 to >20-fold during exercise depending on intensity and, to a lesser extent, duration. Interestingly, noradrenaline does not cross the BBB, yet peripheral adrenaline levels correlate positively with central noradrenaline activity, suggesting that peripheral adrenaline may serve as an indirect marker of central noradrenergic activation (Basso and Suzuki 2017).

Noradrenaline is frequently studied for its dual role in brain and autonomic regulation and as a marker for mood, stress, and cardiovascular function. Its release depends on time and intensity: it peaks during high-intensity exercise near the anaerobic threshold. This is known as the “catecholamine threshold” (Podolin et al 1991; Schneider et al 1992). However, more recent studies emphasize that circulating catecholamine concentrations increase with exercise intensity in a nonlinear manner, with a marked rise at moderate to high intensities (Hackney 2006; Zouhal et al 2008), reflecting enhanced sympathetic activation with a strong correlation between lactate accumulation and adrenaline levels (eg, Schwarz and Kindermann 1990; Zouhal et al 2008). Furthermore, exhaustive or interval training elicits increases in noradrenaline levels up to 10-fold (Lehmann et al 1981). Continuous progressive exercise without breaks also leads to noradrenaline accumulation.

Long-term exercise alters the catecholamine response: after a 10-wk endurance program, trained individuals showed greater noradrenaline responses at 65%–85% VO₂max (Greiwe et al 1999), supporting training adaptations in the sympathoadrenal axis. Furthermore, some studies found significantly lower noradrenaline responses in untrained individuals (Silverman and Mazzeo 1996; Kjær 1998). The concept of the “sports adrenal medulla” is characterized by increased adrenal volume and catecholamine secretion following training (Kjær 1998; Zouhal et al 2008). This may contribute to the enhanced performance capacity observed in trained individuals.

Environmental and psychological stress modulates catecholamine levels. For example, noradrenaline and adrenaline were significantly higher during competitive running vs noncompetitive trials (Karsai et al 2023), whereas flow states were more frequent in noncompetitive settings. Flow is a mental state of deep focus and absorption—marked by a balance between challenge and skill, reduced self-awareness, and a distorted sense of time—often occurring during fully engaging activities such as running (Nakamura and Csikszentmihalyi 2009). Flow has been proposed as one of the core experiential components of the runner’s high, alongside euphoria, anxiolysis, and analgesia (Dietrich and McDaniel 2004). Both states share features such as deep absorption, reduced self-referential processing, and altered time perception (Nakamura and Csikszentmihalyi 2009). However, while flow can occur across a range of goal-directed activities, the runner’s high is specifically linked to prolonged endurance exercise. These findings align with the transient hypofrontality theory, which posits that flow arises from decreased prefrontal activity, allowing for automated and goal-directed performance (Karsai et al 2023). Therefore, excess stress and catecholamine release might impede the achievement of flow during exercise.

Catecholamine levels drop rapidly after exercise; noradrenaline levels approach baseline within 15 min postexhaustion (Holmqvist et al 1986), which may contribute to a postexercise sedative effect as sympathetic tone declines. A 35% decrease in adrenaline within just 1 min postexercise has also been reported, reflecting the rapid clearance of these molecules (Zouhal et al 2008).

### Noradrenaline, Adrenaline, and eCBs in Humans and Animals

The influence of adrenaline and noradrenaline on the eCB system remains an area of ongoing research. To date, only 1 study has examined this connection in humans and reported a correlation between noradrenaline and AEA during alpine hiking (Feuerecker et al 2012). Building on this, further evidence from animal studies indicates that noradrenaline can modulate eCB signaling in contexts such as anxiety (Bellocchio et al 2013), nociception (Suleymanoglu et al 2024), stress adaptation, and neuroplasticity (Haj-Dahmane and Shen 2014). These findings suggest that noradrenergic activity may influence eCB tone and vice versa, particularly in emotionally or physiologically demanding situations such as endurance exercise. However, a clear interaction between the eCB system and adrenaline or noradrenaline has not been systematically characterized in the context of endurance exercise.

In summary, noradrenaline and adrenaline are key neuromodulators during endurance exercise, linking metabolic demand to arousal, attention, and stress regulation. Their elevation supports performance but may also inhibit flow states under high stress. Postexercise declines in catecholamines may contribute to the anxiolytic and calming effects of endurance activity, highlighting their dual role in activating and restoring neural balance.

## Dopamine

Dopamine is a key neurotransmitter involved in movement, reward, motivation, and cognition. It operates through distinct pathways, such as the nigrostriatal, mesolimbic, and mesocortical pathways, that support functions ranging from motor control to reinforcement learning and executive processing (Alcaro et al 2007; Harsing 2008). Dopamine exerts its effects primarily via D1-like (D1, D5) and D2-like (D2, D3, D4) receptors. Its synaptic availability is tightly regulated by dopamine transporters and by enzymatic degradation via monoamine oxidase and catechol-*O*-methyltransferase into the metabolite homovanillic acid (Beaulieu and Gainetdinov 2011).

It plays a central role in the brain’s reward system by encoding reward prediction errors and reinforcing goal-directed behavior, thereby shaping motivation, pleasure, and learning from positive outcomes and negative reward prediction errors (Hollerman and Schultz 1998; Schultz 2016). Importantly, dopamine cannot cross the BBB, meaning that peripheral blood samples offer limited insight into central signaling.

Dopamine interacts bidirectionally with other monoamines such as noradrenaline and 5-HT. Two studies found an increase in dopamine β-hydroxylase after exercise, an enzyme that converts dopamine into noradrenaline in the adrenal glands (Wooten 1973; Peronnet et al 1985). Thus, dopamine β-hydroxylase activity serves as a marker for exercise-induced sympathetic activation in blood, as well as for physical and psychological stress. Furthermore, homovanillic acid was elevated following moderate- and high-intensity endurance exercise (Kendler et al 1983). However, it should be considered that dopamine is also produced in peripheral tissues, including skeletal muscle, visceral organs, and the vascular system, such that peripheral homovanillic acid turnover does not exclusively reflect central dopaminergic activity (Lambert et al 1993).

Since dopamine cannot cross the BBB, neuroimaging studies are required to investigate central dopaminergic activity. Two studies found higher D2 receptor activity in the brain in physically active adults (Dang et al 2017; Jonasson et al 2019). However, 1 PET study failed to detect activation in the putamen, a part of the striatum, after 30 min of treadmill running at ~85% of maximal heart rate, just below the lactate threshold (Wang et al 2000). The authors hypothesized that the exercise stimulus may have been insufficient in intensity or duration to elevate central dopamine levels. Moreover, the timing of the PET scan may have missed any brief dopamine spikes during exercise, capturing only postexercise effects. This highlights a key methodological concern: as imaging protocols typically involve a delay of several minutes to an hour postexercise, such scans may reveal only postexercise changes and miss acute brain responses during physical activity.

### Dopamine and eCBs in Animal Studies

As human studies are scarce, animal studies provide additional insights into the neuromodulatory effects of dopamine during endurance sports. A rodent study found that voluntary exercise and increased effort to obtain food depend on D1 receptor activation, whereas D2 activation had the opposite effect (Walle et al 2024). D1 stimulation and D2 inhibition were associated with increased activity and weight loss. Mechanistically, these findings align with Dohnalová et al (2022), who showed that the gut microbiota can boost exercise performance via dopaminergic modulation. In their study, microbiome-derived eCB metabolites activated TRPV1-positive sensory neurons, leading to increased dopamine release in the ventral striatum—a key region in the reward system. This activation enhanced running motivation and endurance. Disrupting the microbiome, blocking eCB receptors, ablating spinal sensory neurons, or inhibiting dopamine abolished the effect, underscoring dopamine’s role as a final effector in the gut-brain axis regulating voluntary exercise.

Taken together, current evidence indicates that endurance exercise activates the dopaminergic system, including the brain’s reward center. However, due to methodological challenges in measuring central dopamine activity in humans during exercise, it remains difficult to clearly describe the underlying processes. Future analytic approaches in humans are needed to better evaluate dopaminergic activation and its link to reward perception during physical activity.

## Discussion

This review has outlined how endurance exercise elicits a complex pattern of neuromodulatory and neuroendocrine responses that contribute to the runner’s high (Table 1). Among the neuromodulatory systems engaged by endurance exercise, the eCB system appears to play a central and integrative role. eCBs—particularly AEA and, to a lesser extent, 2-AG—increase during moderate-intensity exercise and are linked to features of the runner’s high, including euphoria and anxiolysis (Desai et al 2021; Siebers et al 2021). Beyond their direct affective effects, eCBs interact with other neuromodulators involved in exercise responses (Matei et al 2023). β-endorphins, which peak during high-intensity or prolonged exercise, primarily contribute to pain modulation and stress regulation, potentially complementing eCB-mediated affective regulation. Leptin integrates the metabolic state with motivation and reward and may interact with eCB signaling to enhance locomotor drive and reward sensitivity, particularly under conditions of energy deficit. In addition, 5-HT and BDNF contribute to the antidepressant and cognitive benefits of exercise, while noradrenaline and dopamine support arousal, motivation, and reinforcement learning within this interconnected neuromodulatory network. As the runner’s high represents a subjective experience that manifests only in a subset of individuals (Hinton and Taylor 1986; Siebers et al 2023), identifying a consistent and reproducible biological pattern underlying this phenomenon remains challenging. Interindividual variability further complicates detection, as mood changes may be subtle or not consciously perceived in some individuals. Nevertheless, recent studies have provided converging evidence for exercise-induced mood enhancement: a meta-analysis examining affective responses to physical activity reported improvements in mood, reduced stress, and anxiolytic effects, with small to moderate effects observed for eCBs and BDNF within 1 h after a single bout of moderate to vigorous aerobic exercise (Schaumberg et al 2025). Descriptive analyses further identified that the most consistent associations between biomarkers and outcomes were for eCBs in relation to affective measures. Studies therefore aim to capture this transient and elusive state at the time when it occurs to enable systematic scientific classification. This represents a substantial methodological challenge, particularly given the distinct influence of mindset and expectancy effects (Hinton and Taylor 1986; Dietrich and McDaniel 2004).

**Table 1.** Neuromodulators and Hormones Associated with Endurance Exercise and Features of the Runner’s High.

Neuromodulator/Hormone	Peak Condition during Exercise in Blood (Timing, Duration, and Intensity)	Psychological/Cognitive Effects
Endocannabinoids	Rises during moderate to vigorous exercise (~70%–85% maximal heart rate); remains elevated for up to ~30 min postexercise	Anxiolysis, mood enhancement, analgesia, flow, altered time perception, stress resilience
β-Endorphin	Peaks after prolonged exercise (≥1 h running above 60% VO₂max) or during high-intensity exercise above the lactate threshold	Analgesia, reward, anxiolysis, sedation
Leptin	Declines after ~60 min of endurance exercise or energy expenditure exceeding ~800 kcal; sustained reduction with prolonged activity	Low levels: increased motivation for physical activity, enhanced reward sensitivity, indicator of sports addiction
BDNF	Increases after 20–90 min of aerobic exercise at 40%–60% VO₂max; stronger and longer-lasting effects with regular training	Improves memory, neuroplasticity, mood regulation, fear extinction, stress resilience
Serotonin	Peaks at 85%–90% of maximal heart rate; cortical levels remain elevated for hours to days with regular training	Mood regulation, central fatigue modulation, cognitive enhancement, antidepressant effects
Noradrenaline/adrenaline	Plasma levels rise steeply near maximal effort (at or above ventilatory threshold); transient elevation during and shortly after exercise	Increases arousal, attention, cardiovascular drive; may inhibit flow state
Dopamine	Increases during sustained exercise; peak likely after ~60 min, followed by decline with exhaustion; more research needed	Enhances cognition, motivation, reinforcement learning

Overview of selected neuromodulators and hormones affected by endurance exercise, including peak blood conditions (intensity, duration, and timing) and established psychological or cognitive effects.

BDNF, brain-derived neurotrophic factor.

**Figure 4.**
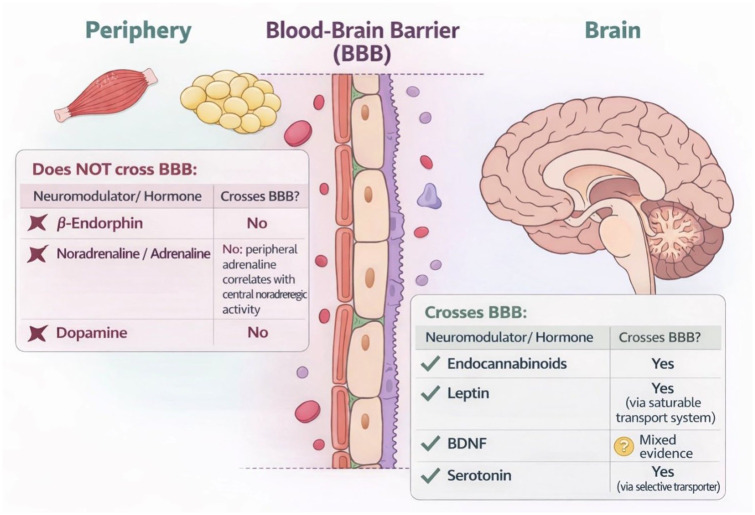
Neuromodulators at the blood-brain barrier (BBB): which compounds cross the BBB? Schematic illustration of the BBB shows selected neuromodulators and hormones according to their ability to cross the barrier. Endocannabinoids, leptin (via a saturable transport system), and serotonin (via selective transporters) can cross the BBB, whereas β-endorphin, dopamine, and peripheral adrenaline and noradrenaline cannot. For brain-derived neurotrophic factor (BDNF), the evidence regarding BBB permeability is mixed.

Peripheral blood samples are often used to infer the role of neuromodulators in the runner’s high (Hinton and Taylor 1986; Siebers et al 2023). A key limitation of this approach concerns whether the measured molecules are able to cross the BBB (Figure 4). Accordingly, this narrative review examines which neuromodulators can cross the BBB. Earlier studies reported elevated peripheral concentrations of several neuromodulators and subsequently hypothesized a central site of activation. However, as indicated most prominently for β-endorphins, many of these molecules do not readily cross the BBB, warranting caution when inferring central nervous system activity from peripheral blood measurements. Importantly, neuromodulators may nevertheless be produced directly within the brain or may access central targets via currently unrecognized transport pathways or indirect signaling mechanisms involving the BBB.

This narrative review represents an initial step toward conceptualizing the neuromodulatory system as an integrated orchestra underlying the runner’s high. Nevertheless, more questions remain than have been answered. For example, the precise sites of eCB production during running, as well as their subsequent degradation, remain incompletely understood. The schematic figure presented in this review constitutes a first attempt to disentangle these processes. Moreover, only a limited number of studies have systematically investigated the interplay of neuromodulatory systems in humans and in animal models, highlighting a substantial gap in the literature (Feuerecker et al 2012; Heyman et al 2012; Ferreira-Vieira et al 2014; Fuss et al 2015; Balsevich et al 2018; Marin Bosch et al 2020; Siebers et al 2021; Dohnalová et al 2022). Future research should aim to delineate the dynamic interactions among these systems in real time. Neuroimaging studies that directly assess central eCB signaling are urgently needed, with careful attention to the timing of measurements to avoid missing transient intraexercise effects. Several PET radiotracers targeting components of the eCB system have already been tested in humans (Burns et al 2007; Terry et al 2010; Normandin et al 2015). A study design involving prolonged endurance exercise (eg, ~2 h, as in Boecker et al 2008) could represent an elegant approach to assess eCB-related signaling in the brain postexercise. Furthermore, studies directly comparing laboratory-based and real-world conditions are lacking, particularly with regard to differences between treadmill running and running in natural environments and their effects on neuromodulators, especially eCBs.

A limitation of this review is that we focused on only a subset of neuromodulators that have been most frequently discussed as contributors to the runner’s high. However, accumulating evidence suggests that additional neuromodulatory systems may be involved: oxytocin (Hew-Butler et al 2008; Jong et al 2015; Wei et al 2015; Wirobski et al 2024), β-phenylethylamine (Szabo et al 2001), insulin-like growth factor 1 (Gatti et al 2012), cortisol (Heyman et al 2012; Caplin et al 2021), irisin (Paoletti and Coccurello 2024), orexin (Tesmer et al 2024), and neuropeptide Y (Kumari et al 2024). These interactions underscore the complexity of exercise-induced neuromodulation and highlight the challenge of attributing affective outcomes to single molecular mediators. Furthermore, a substantial proportion of studies, particularly those investigating the eCB system and its interaction with other neuromodulators, were conducted in rodent models and therefore cannot be directly extrapolated to humans. While there is substantial evidence supporting a central role of eCB signaling within this network, the neuromodulatory “orchestra” likely extends beyond the core players discussed here, with dynamic interactions among multiple systems, which may contribute to the runner’s high.

In conclusion, physical activity, endurance exercise in particular, elicits a robust neuromodulatory response that enhances mood, reduces stress, and promotes mental health. Current evidence indicates that eCBs constitute a central component of this neurochemical orchestra, acting in close concert with opioids, monoamines, neurotrophins, and hormonal systems, all of which play essential and interdependent roles in shaping a unified affective and motivational response. Elucidating the dynamic interplay among these neuromodulators may not only deepen our understanding of the runner’s high but also inform the development of targeted exercise-based interventions for affective disorders.
